# Validation of a Hypoglycemia Risk Stratification Tool Using Data From Continuous Glucose Monitors

**DOI:** 10.1001/jamanetworkopen.2023.6315

**Published:** 2023-03-31

**Authors:** Andrew J. Karter, Melissa M. Parker, Howard H. Moffet, Kasia J. Lipska, James D. Ralston, Elbert S. Huang, Lisa K. Gilliam

**Affiliations:** 1 Division of Research, Kaiser Permanente, Oakland, California; 2 Section of Endocrinology, Department of Internal Medicine, Yale School of Medicine, New Haven, Connecticut; 3 Kaiser Permanente Washington Health Research Institute, Seattle; 4 Section of General Internal Medicine, Department of Medicine, University of Chicago, Chicago, Illinois; 5 Kaiser Northern California Diabetes Program, Endocrinology and Internal Medicine, Kaiser Permanente, South San Francisco Medical Center, San Francisco

## Abstract

This cohort study uses data from continuous glucose monitoring to validate a hypoglycemia risk stratification tool.

## Introduction

The hypoglycemia risk stratification tool^1,2^ was developed to identify patients with diabetes at high risk of emergency department (ED) visits or hospitalizations due to hypoglycemia using electronic health records only. The 12-month rate of hypoglycemia-related ED visits or hospitalizations among patients at high risk was 6.7%, vs 0.3% for those at low or intermediate risk (C statistic = 0.83).^1^ This tool also performed well in 2 external validations among 1 350 938 patients with diabetes (C statistic = 0.79 and 0.81).^1^ Kaiser Permanente Northern California (KPNC) uses this tool to identify patients at high risk (those with ≥3 hypoglycemia-related ED visits or hospitalizations and insulin users with any history of hypoglycemia-related ED visits or hospitalizations). The tool is available as a free online calculator.^3^ To our knowledge, this tool has not been validated against biochemical hypoglycemia based on continuous glucose monitor (CGM) data.

## Methods

This cohort study was conducted among KPNC members with diabetes who shared CGM data with their clinicians and were active CGM users (>70% of the time) for 2 or more weeks during 2020. Participants were classified at baseline as high vs low or intermediate risk using the tool. Each participant’s 2020 CGM data were analyzed using iglu in RStudio Server, version 1.3.1073 (R Group for Statistical Consulting).^4^ Primary outcomes included percentage of time with glucose below 54 mg/dL (to convert to millimoles per liter, multiply by 0.0555) and proportion of patients exceeding the recommended target of less than 1% time with glucose below 54 mg/dL.^5^ Secondary outcomes included percentage of time with glucose below 70 mg/dL and rates of hypoglycemia-related ED visits and hospitalizations during the 12 months after baseline. Statistical analysis was performed from August 2022 to February 2023. Linear regression models were specified to test differences in the continuous variables and binomial generalized linear models with identity link to test differences in proportions (using SAS, version 9.4 [SAS Institute Inc]); *P* values were 2-sided and significant at *P* < .05. This report followed the Strengthening the Reporting of Observational Studies in Epidemiology (STROBE) reporting guideline. The KPNC institutional review board determined that this study was exempt because it involved only secondary research of identifiable private information for which consent is not required.

## Results

Of 2013 eligible CGM users with diabetes (mean [SD] age, 49.9 [17.2] years; 991 [49.2%] women), 421 (20.9%) were classified as high risk for hypoglycemia (Table 1). Participants contributed a mean (SD) of 294.3 (113.6) days of CGM data. The mean percentage of time with glucose below 54 mg/dL was significantly greater among participants at high risk than at low or intermediate risk (0.52% [95% CI, 0.43%-0.62%] vs 0.32% [95% CI, 0.29%-0.34%]; *P* < .001) (Table 2). The percentage of time with glucose below 70 mg/dL was also significantly greater among participants at high risk. The proportion of participants at high risk who exceeded the recommended target of less than 1% of time with glucose below 54 mg/dL was more than double that of participants at low or intermediate risk (65 of 421 [15.4%; 95% CI, 12.0%-18.9%] vs 112 of 1592 [7.0%; 95% CI, 5.8%-8.3%]; *P* < .001). Rates of hypoglycemia-related ED visits or hospitalizations were several-fold higher among participants at high vs low or intermediate risk. Results for each outcome were similar and significant after stratifying by diabetes type, suggesting no algorithmic bias.

**Table 1.** Baseline Characteristics of Participants

Characteristic	No. (%) (N = 2013)
Age, mean (SD), y	49.9 (17.2)
Sex	
Female	991 (49.2)
Male	1022 (50.8)
Race and ethnicity	
African American	131 (6.5)
Asian	168 (8.3)
Latino	217 (10.8)
White	1369 (68.0)
Other or multiracial^a^	92 (4.6)
Unknown	36 (1.8)
Insulin user	1946 (96.7)
Diabetes	
Type 1	1398 (69.4)
Type 2	615 (30.6)
Hypoglycemia risk score	
High	421 (20.9)
Low or intermediate	1592 (79.1)
Days of CGM data contributed, mean (SD)	294.3 (113.6)
ED visit or hospitalization for hypoglycemia prior to baseline	425 (21.1)

Abbreviations: CGM, continuous glucose monitor; ED, emergency department.

^a^ Included Native American (n = 7) and Pacific Islander (n = 9).

**Table 2.** Hypoglycemia-Related Outcomes by Baseline Hypoglycemia Risk Score

Outcome	Baseline hypoglycemia risk score		*P* value
	Low or intermediate (n = 1592)	High (n = 421)	
**Percentage of time with glucose below 54 mg/dL, mean (95% CI), %^a^**			
Overall	0.32 (0.29-0.34)	0.52 (0.43-0.62)	<.001
Type 1 diabetes	0.38 (0.34-0.42)	0.61 (0.54-0.69)	<.001
Type 2 diabetes	0.17 (0.11-0.23)	0.29 (0.17-0.42)	.01
**Participants with ≥1% of time with glucose below 54 mg/dL, No. (%)^b^**			
Overall	112 (7.0)	65 (15.4)	<.001
Type 1 diabetes	100/1093 (9.2)	55/305 (18.0)	<.001
Type 2 diabetes	12/499 (2.4)	10/116 (8.6)	.02
**Percentage of time with glucose below 70 mg/dL, mean (95% CI), %^c^**			
Overall	1.71 (1.60-1.82)	2.30 (2.04-2.56)	<.001
Type 1 diabetes	2.07 (1.93-2.22)	2.68 (2.37-3.00)	<.001
Type 2 diabetes	0.93 (0.81-1.04)	1.30 (0.89-1.70)	.02
**Participants with ED visits and hospitalizations due to hypoglycemia, No./total No. (%)^d^**			
Overall, No. (%)	25 (1.6)	27 (6.4)	<.001
Type 1 diabetes	11/1093 (1.0)	16/305 (5.3)	.001
Type 2 diabetes	14/499 (2.8)	11/116 (9.5)	.02

Abbreviation: ED, emergency department.

SI conversion factor: To convert glucose to millimoles per liter, multiply by 0.0555.

^a^ Results were based on generalized linear regression models for the continuous outcome (percentage of time with glucose below 54 mg/dL); the interaction between diabetes type and hypoglycemia risk score was not statistically significant (*P* = .21).

^b^ Results were based on binomial regression models with identity link for the categorical outcome (≥1% vs <1% of time with glucose below 54 mg/dL); the interaction between diabetes type and hypoglycemia risk score was not statistically significant (*P* = .46).

^c^ Results were based on generalized linear regression models for the continuous outcome (percentage of time with glucose below 70 mg/dL); the interaction between diabetes type and hypoglycemia risk score was not statistically significant (*P* = .21).

^d^ Results were based on binomial regression models with identity link for the categorical outcome (ED visit with primary diagnosis for hypoglycemia or hospitalization with principal diagnosis for hypoglycemia); the interaction between diabetes type and hypoglycemia risk score was not statistically significant (*P* = .43).

## Discussion

This study shows that the tool stratifies risk of hypoglycemia-related ED visits and hospitalizations and percentage of time spent in biochemical hypoglycemia. It was based on patients using CGM from a single health care setting, potentially limiting generalizability. Identifying patients at high risk for hypoglycemia for targeted interventions will be of strategic interest to health plans given the National Committee for Quality Assurance 2023 Healthcare Effectiveness Data and Information Set quality measurement of ED visits for hypoglycemia among older adults with diabetes.^6^ This tool offers a simple approach toward that goal.

## Notes

Supplement.
Data Sharing Statement

## References

[1] Karter AJ, Warton EM, Lipska KJ, . Development and validation of a tool to identify patients with type 2 diabetes at high risk of hypoglycemia-related emergency department or hospital use. JAMA Intern Med. 2017;177(10):1461-1470. doi:10.1001/jamainternmed.2017.3844 28828479 PMC5624849

[2] Karter AJ, Warton EM, Moffet HH, . Revalidation of the hypoglycemia risk stratification tool using *ICD-10* codes. Diabetes Care. 2019;42(4):e58-e59. doi:10.2337/dc18-2154 30765427 PMC6429629

[3] MDCalc. Hypoglycemia risk score. Accessed February 24, 2023. https://www.mdcalc.com/hypoglycemia-risk-score

[4] Broll S, Urbanek J, Buchanan D, . Interpreting blood glucose data with R package iglu. PLoS One. 2021;16(4):e0248560. doi:10.1371/journal.pone.0248560 33793578 PMC8016265

[5] Battelino T, Danne T, Bergenstal RM, . Clinical targets for continuous glucose monitoring data interpretation: recommendations from the international consensus on time in range. Diabetes Care. 2019;42(8):1593-1603. doi:10.2337/dci19-0028 31177185 PMC6973648

[6] National Committee for Quality Assurance. HEDIS MY 2023: see what’s new, what’s changed and what’s retired. Published August 1, 2022. Accessed February 24, 2023. https://www.ncqa.org/blog/hedis-my-2023-see-whats-new-whats-changed-and-whats-retired/

