# Supplementary material for: Validation of a Hypoglycemia Risk Stratification Tool Using Data From Continuous Glucose Monitors
Source: JAMA Netw Open. 2023 Mar 31;6(3):e236315. doi: 10.1001/jamanetworkopen.2023.6315 (PMC10066459; doi:10.1001/jamanetworkopen.2023.6315)
Supplement: Supplement. — Data Sharing Statement [file jamanetwopen-e236315-s001.pdf]

Data Sharing Statement

Karter. Validation of a Hypoglycemia Risk Stratification Tool Using Data From Continuous Glucose Monitors. *JAMA Netw Open*. Published March 31, 2023. doi:10.1001/jamanetworkopen.2023.6315

Data
Data available: No

Additional Information
Explanation for why data not available: IRB approved collection of these confidential health data but did not approve data sharing
